# Novel Flavivirus or New Lineage of West Nile Virus, Central Europe

**DOI:** 10.3201/eid1102.041028

**Published:** 2005-02

**Authors:** Tamás Bakonyi, Zdenek Hubálek, Ivo Rudolf, Norbert Nowotny

**Affiliations:** * University of Veterinary Medicine, Vienna, Austria;; † Szent István University, Budapest, Hungary;; ‡ Institute of Vertebrate Biology ASCR, Brno, Czech Republic;; § United Arab Emirates University, Al Ain, United Arab Emirates

**Keywords:** Rabensburg virus, West Nile virus, Flaviviridae, complete genome analysis, phylogenetic analysis, research

## Abstract

A flavivirus (strain 97-103) was isolated from *Culex pipens* mosquitoes in 1997 following floods in South Moravia, Czech Republic. The strain exhibited close antigenic relationship to West Nile virus (WNV) prototype strain Eg-101 in a cross-neutralization test. In this study, mouse pathogenicity characteristics and the complete nucleotide and putative amino acid sequences of isolate 97-103, named Rabensburg virus (RabV) after a nearby Austrian city, were determined. RabV shares only 75%–77% nucleotide identity and 89%–90% amino acid identity with representative strains of WNV lineages 1 and 2. Another RabV strain (99-222) was isolated in the same location 2 years later; it showed >99% nucleotide identity to strain 97-103. Phylogenetic analyses of RabV, WNV strains, and other members of the Japanese encephalitis virus (JEV) complex clearly demonstrated that RabV is either a new (third) lineage of WNV or a novel flavivirus of the JEV group.

## Abstract 2

Rabensburg virus, isolated from *Culex pipiens* mosquitoes in central Europe, represents a new lineage of West Nile virus or a novel flavivirus of the Japanese encephalitis virus group.

West Nile virus (WNV), a member of the Japanese encephalitis virus (JEV) group within the genus *Flavivirus*, family *Flaviviridae*, is the most widespread flavivirus, occurring in Africa, Eurasia, Australia, and North America. Other members of the JEV group flaviviruses are Cacipacore virus (CPCV), Koutango virus (KOUV), JEV, Murray Valley encephalitis virus (MVEV), Alfuy virus (ALFV), St. Louis encephalitis virus (SLEV), Usutu virus (USUV), and Yaounde virus (YAOV) (*1*). Although initially WNV was considered to have minor human health impact, the human and equine outbreaks in Europe (Romania, Russia, France, Italy), Africa (Algeria, Tunisia, Morocco), and Asia (Israel) within the last 10 years, and especially the virus's emergence and spread in North America since 1999, put it into the focus of scientific interest. The distribution and ecology of WNV, as well as clinical features, pathogenesis, and epidemiology of West Nile disease have been reviewed (*2**–**6*). Phylogenetic analyses showed 2 distinct lineages of WNV strains (which themselves subdivide into several subclades or clusters), isolated in different geographic regions (*7**–**10*).

The presence of WNV in central Europe has been known for some time. Serologic surveys have detected specific antibodies to WNV in several vertebrate hosts in Austria, Czech Republic, Hungary, and Slovakia during the past 40 years, and several virus strains were isolated from mosquitoes, rodents, and migrating birds (*3*). Human cases of West Nile fever were reported in the Czech Republic in 1997 (*11*) and in Hungary in 2003 (*12*). Although these countries are important transit areas or final destinations for migratory birds from the African continent, and hence may play an important role in the circulation and conservation of different WNV strains, genetic information about the strains isolated in central Europe has not been available. We report the complete genome sequence and phylogenetic analyses, as well as antigenic and mouse virulence characteristics, of a unique flavivirus strain (97-103), closely related to WNV, which was isolated by intracranial injection of suckling mice with homogenates of female *Culex pipiens* mosquitoes collected 10 km from Lanzhot, Czech Republic, after a flood in 1997 (*11**,**13**,**14*). The collection site was very close to the Czech-Austrian border, ≈2 km from the small Austrian town of Rabensburg. Consequently, the isolate 97-103 was later tentatively called Rabensburg virus (RabV). Another antigenically identical or very closely related strain (99-222) was isolated from *Cx. pipiens* mosquitoes in the same location 2 years later (*14*).

## Methods

Isolates 97-103 (passage 5 in suckling mouse brain [SMB]) and 99-222 (passage 4 in SMB) were freeze-dried in October 2000 (*14*). Viral RNA was extracted from 140 mL of virus resuspended in diethylpyrocarbonate (DEPC)-treated water, using the QIAamp viral RNA Mini Kit (Qiagen, Hilden, Germany), according to the manufacturer's instructions. For amplification of the complete genome, oligonucleotide primers were designed with the help of the Primer Designer 4 for Windows 95 program (Scientific and Educational Software, version 4.10) and were synthesized by GibcoBRL Life Technologies, Ltd. (Paisley, Scotland, UK). A complete list of the 35 primers used in reverse transcription–polymerase chain reaction (RT-PCR) and sequencing reactions is available upon request. Reverse transcription and amplification were performed with a continuous RT-PCR method with the Qiagen OneStep RT-PCR Kit (Qiagen) following the manufacturer's instructions. Reverse transcription (at 50°C for 30 min) was followed by a denaturation step at 95°C for 15 min, and 40 cycles of amplification (94°C for 40 s, 57°C for 50 s, 72°C for 1 min). Reactions were completed by a final extension for 7 min at 72°C, and the amplicons were kept at 4°C until electrophoresis was carried out. The reactions were performed in a Perkin-Elmer GeneAmp PCR System 2400 thermocycler (Perkin-Elmer Corp., Wellesley, MA, USA). After RT-PCR, the amplicons were electrophoresed in agarose gel, stained with ethidium bromide, and bands were visualized under UV light. Gels were photographed with a Kodak DS Electrophoresis Documentation and Analysis System (Eastman Kodak Company, New Haven, CT, USA). Product sizes were determined with reference to a 100 – bp DNA Ladder (Promega, Madison, WI, USA). Fluorescence-based direct sequencings were performed in both directions on the PCR products with the ABI Prism Big Dye Terminator cycle sequencing ready reaction kit (Perkin-Elmer) and an ABI Prism 310 genetic analyzer (Perkin-Elmer) automated sequencing system (*15*).

The nucleotide sequences were identified by BLAST search against GenBank databases and were compiled and aligned with the help of the Align Plus 4 for Windows 95 (Scientific and Educational Software, version 4.00) and ClustalX Multiple Sequence Alignment (version 1.81) programs. Phylogenetic analysis was performed with the Phylogeny Inference Program Package (PHYLIP) version 3.57c. Distance matrices were generated by the Fitch program, with a translation/transversion ratio of 2.0. Phylogenetic trees were delineated by using the TreeView (Win32) program version 1.6.6.

## Results

Both virus strains were identified as WNV by complement fixation and neutralization tests (*11**,**13*). Strain 97-103 was compared antigenically in detail with the Egyptian Eg-101 topotype strain of WNV (*16*), a representative of WNV lineage 1 (clade 1a). In plaque-reduction cross-neutralization tests (PRNT) with homologous and heterologous antisera (produced by injection of ICR mice with 3 intraperitoneal doses at weekly intervals), the serum raised against Eg-101 neutralized both the homologous virus and 97-103 at a titer of 512, while the strain 97-103 specific serum was effective against strain Eg-101 only at a titer of 64, although it neutralized the homologous virus at 512. The average 4-fold difference in cross-PRNT titers indicates certain antigenic heterogeneity of the 2 strains, and the 97-103 isolate was therefore regarded as "a subtype of WNV" (*14*).

Virulence of RabV strains 97-103 and 99-222 was determined by intracranial and intraperitoneal injection of specific-pathogen-free (SPF) outbred ICR mice. Central nervous system symptoms (e.g., pareses of hind limbs) developed in suckling mice, which died 7–15 days after intracranial injection (Table 1). Adult mice did not show any clinical symptoms and survived the experimental infection. On the other hand, the WNV topotype strain Eg-101 caused fatal illness in intracranially injected mice, killing them within 4 to 6 days after infection, regardless of their age (*11**,**13*). After intraperitoneal injection, strain Eg-101 killed all suckling mice but a <10% of adult mice; RabV strains 97-103 and 99-222 killed approximately one third of suckling mice, and the average survival time was 11 days (range 10–14 days). Thus, both Rabensburg virus strains exhibit clearly lower virulence for mice than the Egyptian WNV topotype strain. In addition, average survival time of suckling ICR mice injected intracranially with RabV was significantly longer than with strain Eg-101.

**Table 1:** Survival time (days) of suckling mice injected intracranially with Rabensburg virus isolates 97-103 and 99-222

Suckling mouse brain (SMB) passage no.	Strain 97-103		Strain 99-222	
	Average survival time	Range	Average survival time	Range
SMB_0_*	12.1	12–13	12.2	9–15
SMB_1_	8.5	7–10	11.8	11–13
SMB_2_	8.5	7–11	10.0	9–11
SMB_3_	8.1	7–9	8.7	7–10

*Represents the original mosquito suspension during virus isolation attempts.

The genome of strain 97-103 Rabensburg virus (RabV) was investigated by RT-PCR and subsequent direct sequencing of the amplicons. Initially, oligonucleotide primers designed on the consensus sequences of linage 1 and 2 WNV strains were applied to the viral nucleic acid of RabV. On the basis of the sequence information obtained from these PCR products, specific primer pairs were designed to produce overlapping amplicons covering the entire genome. The RT-PCR products were sequenced, and the sequences were compiled, resulting in a 10,972 – nucleotide (nt–) sequence that represented the complete genome of the virus. The sequence was identified by BLAST search against GenBank databases. The highest identity rates of RabV to other flaviviruses (78%–90%) were found with certain regions of WNV strains of lineage 1 and 2.

From the second isolate (99-222), 5 genomic regions have been amplified and sequenced so far, showing a total of 3656 nt. They represent partial coding sections from the core (C), anchored C, premembrane (PreM), and membrane (M) protein regions (between nucleotide positions 117 and 752); NS3 protein region (between nucleotide positions 5294 and 5536, and between nucleotide positions 5565 and 6343); NS4b and NS5 regions (between nucleotide positions 7321 and 8112); and NS5 protein region (between nucleotide positions 9095 and 10305). Partial sequence analysis of isolate 99-222 showed >99% identity to 97-103. Aligned to strain 97-103, only a few nucleotide substitutions were observed, in the following positions: C_609_ to T; C_720_ to A; G_5727_ to A (resulting in an amino acid change Met to Ile); T_5910_ to C (resulting in an amino acid change Ile to Thr); T_5961_ to C; C_9630_ to A; and G_9843_ to T.

Similar to other flaviviruses (*17*), the nucleotide sequence of RabV contains l open reading frame (ORF) encoding the viral proteins as a large polyprotein precursor. The ORF starts at nucleotide position 97, and codes for a 3,433-amino acid (aa) polypeptide. The putative amino acid sequence of the polyprotein precursor gene of RabV 97-103 has been translated; based on the amino acid alignment with WNV, the putative mature proteins, conserved structural elements, and putative enzyme motifs were localized. The anchored C protein is located between nt 97 and 465; within this region, the C protein is located between nt 97 and 411. The PreM protein is encoded from nt 466 to nt 966, with the M protein between nt 742 and 966. The envelope (E) protein is encoded between nucleotide positions 967 and 2469, followed by the nonstructural proteins NS1 (nt 2470–3525), NS2a (nt 3526–4218), NS2b (nt 4219–4611), NS3 (nt 4612–6468), NS4a (nt 6469–6846), 2K (nt 6847–6915), NS4b (nt 6916–7680), and NS5 (nt 7681–10395), respectively. Amino acid identities with WNV were found at the known conserved positions (i.e., Cys residues involved in intramolecular bonds in the E and NS1 protein, putative integrin binding motif of the E protein, catalytic triad and substrate binding pocket of the trypsin-like serine protease, RNA helicase motif of the NS3 protein, and RNA-dependent RNA polymerase motif of the NS5 protein 15; ).

To investigate the phylogenetic relationship of RabV to other WNV isolates, multiple nucleotide and putative amino acid sequence alignments were made involving 16 WNV strains (listed in Table 2). Although several complete WNV nucleotide sequences from previously published studies (*10**,**18*) have been deposited in the GenBank databases, only selected representatives of lineages and clades have been included in our alignments, in order to obtain more precise and demonstrative trees.

**Table 2:** Sequences of West Nile virus (WNV) strains and other members of the Japanese encephalitis virus group used for phylogenetic analyses

Virus name	Code	Accession no.*	Isolation			
			Year	Host	Geographic origin	WNV lineage, clade
WNV HNY1999	NY99a	AF202541	1999	Human	New York	1a
WNV NY99flamingo38299	NY99b	AF196835	1999	Flamingo	New York	1a
WNV IS98STD	Is98	AF481864	1998	Stork	Israel	1a
WNV Italy1998Equine	It98	AF404757	1998	Horse	Italy	1a
WNV RO9750	Ro96	AF260969	1996	*Culex pipiens*	Romania	1a
WNV VLG4	Rus99a	AF317203	1999	Human	Volgograd	1a
WNV LEIV-Vlg99-27889	Rus99b	AY277252	1999	Human	Volgograd	1a
WNV PaH001	Tu97	AY268133	1997	Human	Tunisia	1a
WNV PaAn001	Fr00	AY268132	2000	Horse	France	1a
WNV Eg101	Eg51	AF260968	1951	Human	Egypt	1a
WNV Chin-01	Chin01	AY490240	Unknown	Unknown†	China	1a
WNV Kunjin MRM61C	Kunjin	D00246	1960	*Cx. annulirostris*	Australia	1b
WNV Sarafend	Sarafend	AY688948		Laboratory strain		2
WNV B956 (WNFCG)	Ug37	M12294	1937	Human	Uganda	2
WNV LEIV-Krnd88-190	Rus98	AY277251	1998	*Dermacentor marginatus*	Caucasus	4†
Rabensburg virus (97-103)	RabV	AY765264	1997	*Cx. pipiens*	Czech Republic	3†
Japanese encephalitis virus	JEV	NC_001437	–	–	–	–
Murray Valley encephalitis virus	MVEV	NC_000943	–	–	–	–
Usutu virus	USUV	AY453411	–	–	–	–
Saint Louis encephalitis virus	SLEV	*AF013416*	–	–	–	–
Alfuy virus	ALFV	*AF013360*	–	–	–	–
Cacipacore virus	CPCV	*AF013367*	–	–	–	–
Koutango virus	KOUV	*AF013384*	–	–	–	–
Yaounde virus	YAOV	*AF013413*	–	–	–	–

*Partial nucleotide sequences (NS5 protein region) are indicated in italics. †, tentative speciation.

**Figure 1:**
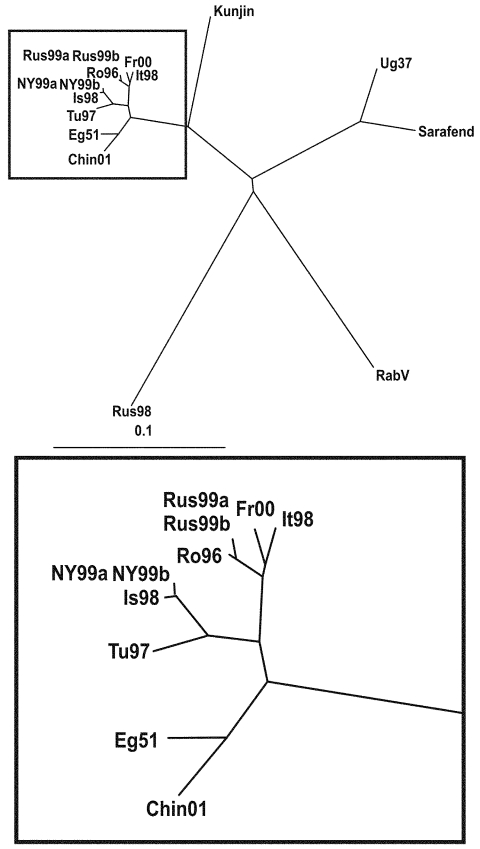
Phylogenetic tree illustrating the genetic relationship between selected West Nile virus strains based on their complete genome sequences. Bar on the left demonstrates the genetic distance. (Abbreviations and accession numbers are listed in Table 2.)

RabV exhibited 73%–77% nucleotide identity rates to the different WNV strains (Table 3). The relationships between the strains are demonstrated in Figure 1. The 2 lineages of WNV are obviously separated in the tree. Clade 1a viruses form a tight cluster with close genetic relationship among the members. Kunjin virus, the representative of clade 1b, appears as a separate branch of lineage 1. Unfortunately, no complete genome sequence information is available on clade 1c (Indian strains); thus, they are not represented in the tree. The prototype Uganda strain B956 (WNFCG) of lineage 2 is grouped together with the Sarafend strain, a laboratory strain with uncertain origin and passage history. Two viruses proved to be clearly distinct with significant genetic distances to all other WNV strains and also from each other: RabV and strain LEIV-Krnd88-190 (in the phylogenetic trees designated Rus98). The latter virus was isolated from *Dermacentor marginatus* ticks in the northwest Caucasus Mountain valley in 1998 and was regarded as a new variant of WNV (*19**–**21*). Because these 2 viruses differ considerably from all WNV strains, the issue is raised about whether classifying these 2 viruses as separate members of the JEV group might be more appropriate.

**Table 3:** Nucleotide and amino acid identity rates between RabV* and other flaviviruses

Code	WNV lineage and clade	Identity to RabV (%)			
		Nucleotide		Amino acid	
		Complete	Partial†	Complete	Partial‡
NY99a	1a	77	78	90	95
NY99b	1a	77	78	90	95
Is98	1a	77	78	90	95
It98	1a	77	78	90	95
Ro96	1a	77	78	90	95
Rus99a	1a	77	78	90	95
Rus99b	1a	77	78	90	95
Tu97	1a	76	78	90	95
Fr00	1a	77	78	90	95
Eg51	1a	77	78	90	95
Chin01	1a	77	78	90	95
Kunjin	1b	75	77	89	94
Sarafend	2	77	78	90	96
Ug37	2	77	78	90	96
Rus98	4 (speculation)	73	77	87	95
JEV	–	68	74	75	86
MVEV	–	69	74	76	86
USUV	–	68	72	75	83
SLEV	–	–	71	–	78
ALFV	–	–	74	–	88
CPCV	–	–	71	–	79
KOUV	–	–	76	–	90
YAOV	–	–	75	–	87

*RabV, Rabensburg virus; JEV, Japanese encephalitis viru*s*; MVEV, Murray Valley encephalitis viru*s*; USUV, Usutu viru*s*; SLEV, St. Louis encephalitis viru*s*; ALFV, Alfuy virus; CPCV, Cacipacore virus; KOUV, Koutango virus; YAOV, Yaounde virus. †Partial alignment between nucleotide positions 9067 and 10101. ‡Partial alignment between amino acid positions 2991 and 3335.

**Figure 2:**
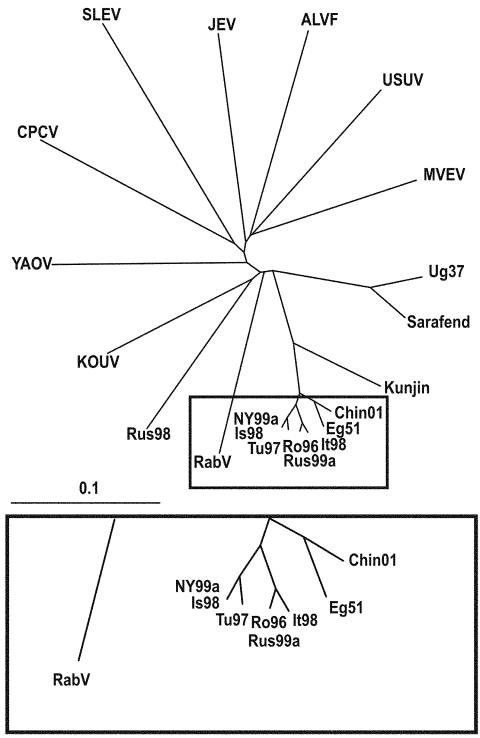
Phylogenetic tree illustrating the genetic relationship between representatives of the Japanese encephalitis virus complex and selected West Nile virus strains based on partial genome sequences of the NS5 protein gene. Bar on the left demonstrates the genetic distance. (Abbreviations and accession numbers are listed in Table 2.)

To elucidate this question, a comprehensive phylogenetic analysis was performed on all representatives of the JEV group. Because only partial common sequence information of the NS5 protein gene region is currently available from SLEV, ALV, CPCV, KOUV, and YAOUV (22), the phylogenetic analysis had to be restricted to this region (Figure 2). Within the investigated genome stretch, RabV showed 77%–78% identity to lineage 1 and 2 WNV strains, 77% identity to strain LEIV-Krnd88-190, and 71%–76% identity to other representatives of the JEV group. In the phylogenetic tree (Figure 2), the separation of the 2 unique strains (RabV and LEIV-Krnd88-190 = Rus98) from WNV is clearly visible. Although RabV exhibits the closest relationship to the WNV representatives, similar identity rates (76%) exist between MVEV and USUV, as well as between JEV and ALFV, and these viruses have been taxonomically classified as separate viruses. The Rus98 virus clusters together with KOUV, a virus isolated originally from a Kemp's gerbil (*Tatera kempi*) in Senegal 1968 and subsequently recovered from other rodent species and several genera of ticks (*Rhipicephalus*, *Hyalomma*, *Alectorobius*) in central Africa (*23*). The Rus98 strain was also isolated from ticks.

The putative amino acid sequence of RabV was also compared with the corresponding sequences of representatives of WNV lineages and clades, as well as with other JEV group viruses on the available polypeptide sequence regions. RabV shared 89%–90% identity on the complete polypeptide precursor region with the WNV strains, 87% identity with the Rus89 strain, and 75%–76% identity with JEV, USUV, and MVEV. The alignments of the partial amino acid sequences of the NS5 region (between aa 2991 and 3335) showed 94%–96% identity rates with the WNV strains, 95% with strain Rus98, and 78%–90% with the other members of the JEV group (Table 3). Phylogenetic trees, based on the amino acid alignments, displayed nearly identical topology to nucleotide sequence–based trees (data not shown). The complete genome sequence of RabV (flavivirus strain 97-103) has been deposited in GenBank under accession no. AY765264.

## Discussion

WNV strains of different lineages exhibit considerable genomic diversity (76%–77% nucleotide identity only). At the same time, WNV is not sharply delimited genomically from the other members of the JEV group. The available partial sequences of the NS5 gene region from other viruses of the group show 71%–76% nucleotide and 78%–90% amino acid identities to WNV strains. The closest relatives of WNV are KOUV and YAOV (*10**,**22**–**24*).

Lineage 1 of WNV comprises strains from several continents and is subdivided into at least 3 clades. In clade 1a, several subclades or clusters are formed by closely related strains, such as strains isolated 40–50 years ago in Europe and Africa; strains isolated 20–30 years ago in Africa; strains isolated within the last 10 years in Europe and Africa; and strains isolated within the last 5 years in the United States and Israel. Clade 1b consists of the Australian isolates (Kunjin), while clade 1c contains strains from India. Lineage 2 is composed of WNV strains that have been isolated, so far exclusively, in the sub-Saharan region of Africa and in Madagascar (*18*). The genetic distance between the 2 lineages is relatively great in contrast to that within some representatives of lineage 1 that were isolated in distant geographic locations and within considerable time intervals. While the viruses in clade 1a share 95.2%–99.9% nucleotide and 99.3%–100% amino acid identity to each other, and also 86.6%–87.8% nucleotide and 97.4%–97.7% amino acid identity to the clade 1b viruses, the overall identity rates between lineage 1 and 2 are only 75.7%–76.8% on nucleotide level and 93.2%–94.0% on amino acid level (*18*), identity rates that resemble those between RabV and either lineage 1 or lineage 2 WNV strains. Besides genomic differences, antigenic variability can be observed in cross-neutralization analyses and monoclonal antibody binding assays (*8**,**18*).

The results of the phylogenetic analyses indicate that viruses closely related to WNV are present in central Europe and southern Russia. Although these viruses have initially been identified as WNV, they can be regarded, on the basis of their genetic distances, either as separate lineages of WNV (RabV: lineage 3; LEIV-Krnd88-190 = Rus98: lineage 4) or as new viruses within the JEV group. The antigenic and biologic differences between RabV and the WNV reference strain Eg-101 also support this opinion. Isolation of RabV in 1997 was obviously not an isolated event; rather, flaviviruses of the RabV type seem to be present or persist in this area, as demonstrated by the isolation of an almost identical virus strain (99-222) 2 years later (*14*). The ecology of RabV needs further investigation. Other unanswered questions concern the pathogenicity and host spectrum of the virus, especially regarding possible human infections.

To summarize, a novel flavivirus strain of unknown human pathogenicity, repeatedly isolated from *Cx. pipiens* mosquitoes in central Europe, has been molecularly characterized, including determination of its complete nucleotide and deduced amino acid sequences. Based on the analysis of the virus and comparison with related viruses including phylogenetic relationships, we suggest that RabV be classified either as a new (third) lineage of WNV or as a novel flavivirus within the JEV group.
